# Errata

**Published:** 1883-08

**Authors:** 


### ERRATA.

When correspondents live at such a distance that it is impossible for us to send them proof sheets to correct, errors will creep in. Usually they are scarce worth notice, save for the annoyance they give to the author, but in Dr. W. D. Miller's article in the June number, are two which affect the scientific accuracy of the paper.

Page 309, line 4, for "c. m.," read "m. m."

Page 306, Fig. 2, for "zone of softened dentine," read "zone of softened non-infected dentine."

Also in Dr. Palmer's paper in the July number, page 357, fifth line from the bottom, for "Dr. R. M. Stenlee," read "Dr. R. M. Streeter."

